# An Out-of-Season Case of Coxsackie B Myocarditis with Severe Rhabdomyolysis

**DOI:** 10.1155/2018/4258296

**Published:** 2018-10-30

**Authors:** Alaap Mehta, Kali Larson, Bindu Sarika Ganapathineedi, Esteffania Villegas, Susmitha Dande, Wahaj Ahmed, Nadew Sebro

**Affiliations:** ^1^Internal Medicine Residency Program, Mount Sinai Hospital, Chicago, IL, USA; ^2^Ross University School of Medicine, Portsmouth, Dominica

## Abstract

A 21-year-old woman was found to have fulminant myocarditis as a result of Coxsackie B infection (a virus shown to exhibit summer-fall seasonality) in mid-December. In this case report, seasonality of enteroviruses is examined, as well as additional factors which may contribute to sporadic cases during winter months. The case report also discusses clinical criteria for endomyocardial biopsy, utility of PCR vs. antibody serological tests, coinfection with multiple serotypes, and rhabdomyolysis in Coxsackie B.

## 1. Background

Winter seasonality is observed commonly in viral infectious diseases due to a variety of proposed mechanisms. Animal and human studies have demonstrated factors such as increased indoor confinement, poor cross ventilation from closed doors and windows, decreased production of vitamin D, impairment of mucociliary clearance, ecological phenomenon such as migration of birds, pathogen-pathogen interaction, and effects of low humidity on hosts and pathogens likely contribute to this phenomenon [1]. On the contrary, enteroviruses such as Coxsackie B have demonstrated prominent summer-fall seasonality due to proposed driving climate factors such as dew point temperature [2]. This patient presented in mid-December with a severe case of acute Coxsackie B myocarditis. Coxsackie is a common cause of acute myocarditis in young patients, accounting for 20–25% of cases annually [3]. Although cases may be asymptomatic, others may present with a fulminant course of acute systolic heart failure due to impaired contractility of the injured myocardium [4]. This case report explores the role of seasonality and additional factors that may increase host susceptibility to enteroviruses during winter months. This report also discusses relevant topics that enrich and inform future care of such patients: clinical criteria for diagnosis of acute viral myocarditis, the development of Coxsackie B-induced rhabdomyolysis, role of PCR vs. antibody in the diagnosis of enteroviruses, and the significance of coinfection with multiple serotypes of Coxsackie B virus.

## 2. Case Presentation

**Figure 1:**
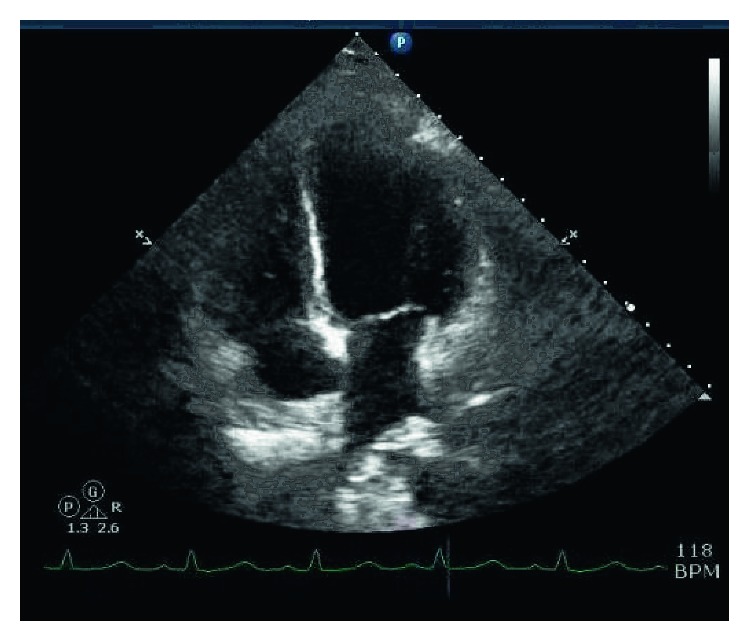
2D echo (four-chamber view) during systole with LVEF of 20–25% (courtesy of Mt. Sinai Hospital Cardiology Department).

A 21-year-old woman with no significant medical history except for treatment for right breast abscess two months prior, presented to the hospital with one week of fever, chills, myalgias, nausea, vomiting, diarrhea, cough, and progressive shortness of breath. The diarrhea was nonbloody and watery in consistency with a frequency of four episodes a day. She denied any chest pain, facial or leg swelling, weakness, headache, or dizziness. She stated her son had an unspecified illness recently that has resolved but denied any other sick contacts. She was found to be febrile with a temperature of 102.0°F, hypotensive with a blood pressure of 82/56 mmHg, and tachycardic at a rate of 149 bpm. A full physical exam was benign except for axillary and cervical lymphadenopathy. Laboratory workup revealed segmented neutrophil predominant leukocytosis, elevated levels of troponin (2.45 ng/ml), BNP (457.2 pg/ml), and d-dimer (6.72 *µ*g/ml). Electrocardiogram (EKG) demonstrated sinus tachycardia with possible left atrial enlargement. Vasopressor support and unfractionated heparin drip were initiated, and the patient was admitted to the intensive care unit. Subsequent imaging showed a low-probability VQ scan for pulmonary embolism, and severe diffuse myocardial hypokinesis with left ventricular ejection fraction (LVEF) of 20–25% without pericardial effusion on 2D Echo (Figure 1).

**Figure 2:**
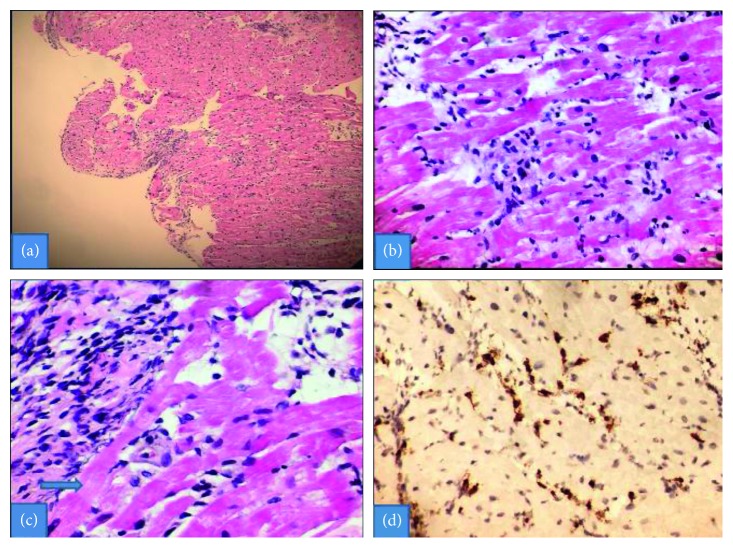
(a) Myocardial biopsy with inflammatory infiltrate; (b) lymphocytic infiltrate; (c) focal myocyte necrosis (arrow); (d) immunohistochemical stain for CD3 highlighting T cells (courtesy of Mt. Sinai Hospital Pathology Department).

While CT abdomen demonstrated generalized lymphadenopathy and mild hepatosplenomegaly, chest X-ray was negative for any acute pathology on admission. Detailed rheumatologic workup was unremarkable, including antinuclear antibody (ANA), double-stranded DNA, antiproteinase 3, antimyeloperoxidase, and C3 and C4 levels. Right heart catheterization was performed and demonstrated elevated wedge pressure, pulmonary arterial pressure, and right ventricular pressure consistent with acute left ventricular failure and secondary pulmonary arterial hypertension. With a presumptive diagnosis of fulminant acute myocarditis, right ventricular endomyocardial biopsy (EMB) was performed disclosing a lymphocytic infiltrate with focal myocyte necrosis along with immunohistochemical stain for CD3 demonstrating presence of T cells (Figure 2).

Serum Polymerase chain reaction (PCR) showed positivity for rhinovirus and Coxsackie viruses and further antibody titers confirmed significantly high but varying titer levels specific to six Coxsackie serotypes: B1 (1 : 32), B2 (1 :16), B3 (1 : 8), B4 (1 : 8), B5 (1 : 64), and B6 (1 : 64). A multidisciplinary team of cardiology, infectious disease, nephrology, and rheumatology initiated treatment with high dose steroid therapy and metoprolol. The hospital course was complicated by worsening acute kidney injury (AKI) and rhabdomyolysis with serum phosphocreatine kinase (CPK) levels as high as 21,867, both of which improved with IV fluid therapy. The patient's clinical condition progressively improved, with almost complete recovery of LVEF to 40–45%. The patient was downgraded to the medical floors and was stable for discharge by hospital day 8. The patient was discharged on lisinopril and metoprolol for compensated heart failure with a plan for close follow-up with cardiology, nephrology, and primary care. Repeat 2D-Echo done one month after discharge revealed complete resolution of systolic function with LVEF of 55–60%.

## 3. Discussion

### 3.1. Myocarditis

Myocarditis is inflammation of the myocardium. Clinically, it can present with symptoms that range from nonspecific symptoms such as fever, myalgia, and palpitations to fulminant hemodynamic collapse and sudden death [5]. Myocarditis can also lead to dilated cardiomyopathy. The wide range of clinical manifestations has made it difficult to determine the true incidence of myocarditis. The Dallas pathological criteria, despite their limitations, present helpful initial diagnostic guidelines to classify myocarditis [5]. Active myocarditis is characterized by an inflammatory cellular infiltrate with evidence of myocyte necrosis, whereas borderline myocarditis demonstrates an inflammatory cellular infiltrate without evidence of myocyte injury [5].

Although a broad array of etiologies have been implicated as causes of myocarditis, adenoviruses and enteroviruses, specifically Coxsackie group B serotypes, have traditionally been perceived as the predominant cause. Hepatitis B and HIV have also been implicated as a cause of myocarditis [5].

History and physical exam findings suggestive of heart failure may provide clues leading to a diagnosis of myocarditis. Additionally, EKG (sinus tachycardia with nonspecific ST-T wave changes), troponin T/I, and creatine kinase, and echocardiogram (global hypokinesis with or without pericardial effusion) could be seen. Obtaining an MRI has recently emerged as a highly sensitive and specific tool for the diagnosis of myocarditis. It has the unique potential to visualize tissue changes and can detect the characteristic changes in myocarditis including intracellular and interstitial edema, capillary leakage, and hyperemia [6].

Endomyocardial biopsy is indicated when giant cell myocarditis or necrotizing eosinophilic myocarditis is suspected. A recently published American Heart Association/American College of Cardiology/European Society of Cardiology joint statement recommends performing EMB in scenarios compatible with fulminant and giant cell myocarditis, and in acute heart failure unresponsive to treatment [6]. Fulminant myocarditis is defined as new onset heart failure of less than two weeks duration associated with hemodynamic compromise. It is a class IB indication for EMB in patients with suspected viral myocarditis [7].

### 3.2. Rhabdomyolysis in Coxsackie B

Rhabdomyolysis from Coxsackie virus is generally rare. When it occurs, Coxsackie virus infections have been associated with a wide spectrum of muscle disorders, ranging from acute nonspecific myalgia to myositis. Though the exact mechanism of viral rhabdomyolysis is still unknown, the end result is destruction of myocytes and the release of toxins into the circulation. Literature review shows that biopsies of patients with rhabdomyolysis with lymphocytic infiltrate support the hypothesis of direct viral invasion [8]. The severity and clinical presentation of rhabdomyolysis also varies from patient to patient, where AKI was documented at relatively lower CPK values, while no renal injury was seen at CPK values as high as 600,000 unit/L. In this patient, CPK values peaked to 21,867 unit/L causing AKI with creatinine 2.23 mg/dl from a normal baseline. Therefore, viral rhabdomyolysis is a diagnosis that needs to be made early and treated aggressively with intravenous fluids due to its unpredictable nature in causing serious complications [9].

### 3.3. Enterovirus Seasonality

Coxsackie viruses are small, acid-stable RNA viruses that are widely present in environmental media. They are commonly excreted by asymptomatic carriers. They are primarily transmitted by feco-oral route, but also through surface contact and airborne droplet nuclei in the minority of cases. Coxsackie infection is commonly seen in children and the male sex. Although their virulence for humans is extremely variable, Coxsackie B infection may cause a wide variety of clinically recognizable diseases, such as pleuritis, myocarditis, pericarditis, pancreatitis, and hepatitis [10]. Coxsackie A virus, on the other hand, is known to cause hand-foot-and-mouth disease (HFMD), hemorrhagic conjunctivitis, and herpangina. Meningitis can be caused by both Coxsackie A and B.

According to NESS (National Enterovirus Surveillance Study), enterovirus detections have prominent summer-fall seasonality, with June–October accounting for 77.9% of reports with known month of specimen collection. Cerebrospinal fluid was the most common specimen type, followed by respiratory and fecal specimens (49.8%, 26.9%, and 13.6%, respectively) [1]. Though the seasonal peak of Coxsackie occurs during summer and fall due to factors like dew point temperatures, some sporadic cases may occur during the winter season due to host factors like the weakened immune system due to vitamin D deficiency, diabetes, and exposure to asymptomatic carriers such as working with children (daycare centers) [2]. In general, the rate of inactivation and extent of survival for viruses depends on virus type, temperature, suspending medium, and other environmental conditions. Climatic factors like low humidity during winter cause small (0.02–0.04 microns) viruses to stay suspended in the air for longer periods of time leading to prolonged exposure and also overcome environmental stress due to antigenic variations [3]. Additionally, some enterovirus serotypes systematically circulate earlier than others in the season. All these environmental, host, and pathogen factors together may be contributing to sporadic cases of coxsackie infections during winter as seen in this patient [5, 11]. A vitamin D level was not checked on this patient, a limitation that would have helped identify a treatable host factor.

### 3.4. Serological Tests

Most acute viral infections do not need molecular diagnosis; however, due to the etiologic association to various diseases like myocarditis, aseptic meningitis, encephalitis, pleurodynia, pericarditis, and diabetes mellitus, identifying a viral etiology can help customize treatment and understand prognosis [11]. Real-time polymerase chain reaction (RT-PCR) is the initial recommended method due to availability of easy sample (nasopharyngeal swab), rapidity, sensitivity, and cost [10, 12]. Other molecular tools like immunohistochemical techniques, ELISA, and in situ hybridization require more time and different samples. EMB samples could also be used for molecular analyses.

This patient had positive antibody titers to six different serotypes of coxsackie B, 1 through 6. This could represent coinfection with various serotypes concurrently (true positive), a heterotypic antibody response (false positive) to infection by a single serotype, or past infections by different serotypes at various time periods (old and new infections) [13]. There are reports that entertain the plausibility of enterovirus coinfection leading to synergies in the pathogenic mechanism [14].

## 4. Conclusion

This case calls for increased level of awareness of the seasonality in specific viral infections. In particular, it calls for further investigation into factors that may contribute to the uncharacteristic occurrences of sporadic cases of enteroviruses during the winter months. Additionally, this case report emphasizes the need for early diagnosis of acute rhabdomyolysis in Coxsackie B infections to prevent subsequent acute kidney injury. It also serves as a reminder that fulminant myocarditis (new onset heart failure of less than two weeks duration associated with hemodynamic compromise) is a class IB indication for EMB in patients with suspected viral myocarditis. Finally, it highlights the utility, sensitivity, and cost-effectiveness of RT-PCR and recognizes the benefit of additional studies for specific etiologic diagnosis.

## References

[1] Altizer S., Dobson A., Hosseini P., Hudson P., Pascual M., Rohani P. (2006). Seasonality and the dynamics of infectious diseases. *Ecology Letters*.

[2] Pons-Salort M., Oberste M. S., Pallansch M. A. (2018). The seasonality of nonpolio enteroviruses in the United States: patterns and drivers. *Proceedings of the National Academy of Sciences*.

[3] Kim K. S., Hufnagel G., Chapman N. M., Tracy S. (2001). The group B coxsackieviruses and myocarditis. *Reviews in Medical Virology*.

[4] Feldman A. M., McNamara D. (2000). Myocarditis. *New England Journal of Medicine*.

[5] Magnani J. W., Dec G. W. (2006). Myocarditis. *Circulation*.

[6] Shauer A., Gotsman I., Keren A. (2013). Acute viral myocarditis: current concepts in diagnosis and treatment. *Israel Medical Association journal*.

[7] McCarthy R. E., Boehmer J. P., Hruban R. H. (2000). Long-term outcome of fulminant myocarditis as compared with acute (Nonfulminant) myocarditis. *New England Journal of Medicine*.

[8] Singh U., Scheld W. M. (1996). Infectious etiologies of rhabdomyolysis: three case reports and review. *Clinical Infectious Diseases*.

[9] Patel S., Mulyala R., Katta N. (2014). Unusual presentation of coxsackie B rhabdomyolysis: case report and literature review. *American Journal of Hospital Medicine*.

[10] Harvala H., Broberg E., Benschop K. (2018). Recommendations for enterovirus diagnostics and characterisation within and beyond Europe. *Journal of Clinical Virology*.

[11] Tracy S., Chapman N. M., Mahy B. W. (2013). *The Coxsackie B Viruses*.

[12] Rotbart H. A., Ahmed A., Hickey S. (1997). Diagnosis of enterovirus infection by polymerase chain reaction of multiple specimen types. *Pediatric Infectious Disease Journal*.

[13] Klingel K., Sauter M., Bock C. T., Szalay G., Schnorr J.-J., Kandolf R. (2004). Molecular pathology of inflammatory cardiomyopathy. *Medical Microbiology and Immunology*.

[14] Yang F., Du J., Hu Y. (2011). Enterovirus coinfection during an outbreak of hand, foot, and mouth disease in Shandong, China. *Clinical Infectious Diseases*.

